# A biocompatible vascularized graphene oxide (GO)-collagen chamber with osteoinductive and anti-fibrosis effects promotes bone regeneration *in vivo*

**DOI:** 10.7150/thno.42006

**Published:** 2020-02-03

**Authors:** Huimin Fang, Chao Luo, Shaokai Liu, Muran Zhou, Yuyang Zeng, Jinfei Hou, Lifeng Chen, Shan Mou, Jiaming Sun, Zhenxing Wang

**Affiliations:** 1 Department of Plastic Surgery, Union Hospital, Tongji Medical College, Huazhong University of Science and Technology, Wuhan 430022, China.; 2 Wuhan Clinical Research Center for Superficial Organ Reconstruction, Wuhan 430022, China

**Keywords:** microenvironment, graphene oxide, tissue engineering chamber, *in situ* bone regeneration, bone mesenchymal stem cells

## Abstract

The survival of transplanted cells and tissues in bone regeneration requires a microenvironment with a vibrant vascular network. A tissue engineering chamber can provide this *in vivo*. However, the commonly used silicone chamber is biologically inert and can cause rejection reactions and fibrous capsule. Studies have revealed that collagen is highly biocompatible and graphene oxide (GO) could regulate osteogenic activity *in vivo*. Besides, GO can be cross-linked with natural biodegradable polymers to construct scaffolds.

**Methods**: A vascularized GO-collagen chamber model was built by placing vessels traversing through the embedded tissue-engineered grafts (osteogenic-induced bone mesenchymal stem cells -gelatin) in the rat groin area. Osteogenic activity and inflammatory reactions were assessed using different methods including micro-CT scanning, Alizarin red staining, and immunohistochemical staining.

**Results**: After one month, *in vivo* results showed that bone mineralization and inflammatory responses were significantly pronounced in the silicone model or no chamber (control) groups. Vascular perfusion analysis confirmed that the GO-collagen chamber improved the angiogenic processes. Cells labeled with EdU revealed that the GO-collagen chamber promoted the survival and osteogenic differentiation of bone mesenchymal stem cells.

**Conclusion**: Overall, the novel biocompatible GO-collagen chamber exhibited osteoinductive and anti-fibrosis effects which improved bone regeneration *in vivo*. It can, therefore, be applied to other fields of regenerative medicine.

## Introduction

Despite considerable progress in bone tissue engineering, practical approaches for creating a suitable microenvironment for better survival of tissue-engineered bone grafts *in vivo* have not been developed 1,2. In the initial phase after implantation, insufficient vascularization induces cell death due to nutrient deprivation 3. Also, severe foreign body reactions trigger macrophage aggregation and fibrous formation, which negatively affect the survival rate of the grafted tissues 4. Biological activity of cell scaffolds plays a significant role in the survival and differentiation of transplanted tissues 5,6. Therefore, a microenvironment with a vibrant vascular network and osteoinductive/anti-fibrosis effects is essential for the survival of a tissue-engineered bone graft.

Tissue engineering chamber is an *in vivo* surgical device that provides a relatively isolated and vascularized environment for graft tissues or cells 7. The chamber wall provides mechanical support for inner grafts, reduces the oppression from surrounding tissue, and prevents macrophage phagocytosis. Angiogenic sprouting stems from the original vessels and progressively develops into a complex vascular network pervading the entire tissue 8. Various cells and tissue types that are difficult to culture *in vitro*, such as islet cells, liver progenitor cells, cardiac tissue, and adipose tissue, can survive and differentiate in tissue engineering chambers *in vivo* 9-11. Several studies have demonstrated the *in vivo* bone regeneration potential of various osteogenic biomaterials 12-15. However, only a few studies have evaluated the performance of the tissue engineering chamber model in bone regeneration or have applied biomaterials in the construction of a tissue engineering chamber.

Currently used tissue engineering chambers are mainly made of plastic and silicone, which require a second operation. Repeated operations activate inflammatory cells and cytokines, leading to inflammatory reactions and fibrous capsule formation 16. Moreover, bioinert materials lack the differentiation-induced biological activity to support differentiating stem cells 17. These drawbacks hinder the application of the tissue engineering chamber model. Therefore, biomaterials with excellent biocompatibility and biological activity are needed for the construction of the tissue engineering chamber.

As a classical tissue engineering scaffold, collagen has been widely used in tissue engineering because of its low immunogenicity, porous structure, good permeability, biocompatibility, and biodegradebility. However, the poor mechanical properties of collagen scaffolds limit their applications 18. Graphene oxide (GO) is a chemically modified graphene containing oxygen functional groups with favorable chemical and biological properties 19-23. After intravenous injection, GO nanoparticles are eliminated from the body through the hepatobiliary route 24. Previous studies have confirmed that GO supports the growth and osteogenic differentiation of stem cells 25,26. The compressive strengths of collagen-based scaffolds can be increased by cross-linking with graphene oxide 27-29. GO-collagen is a biocompatible material with negligible cytotoxicity, and various cell types can survive and differentiate in this scaffold 30,31. The GO-collagen tissue engineering chamber has higher biocompatibility with osteogenic activity and anti-fibrosis potential when compared to traditional silicone implants which tend to cause the formation of fibrous capsule or even capsular contracture 32,33.

**Figure 1:**
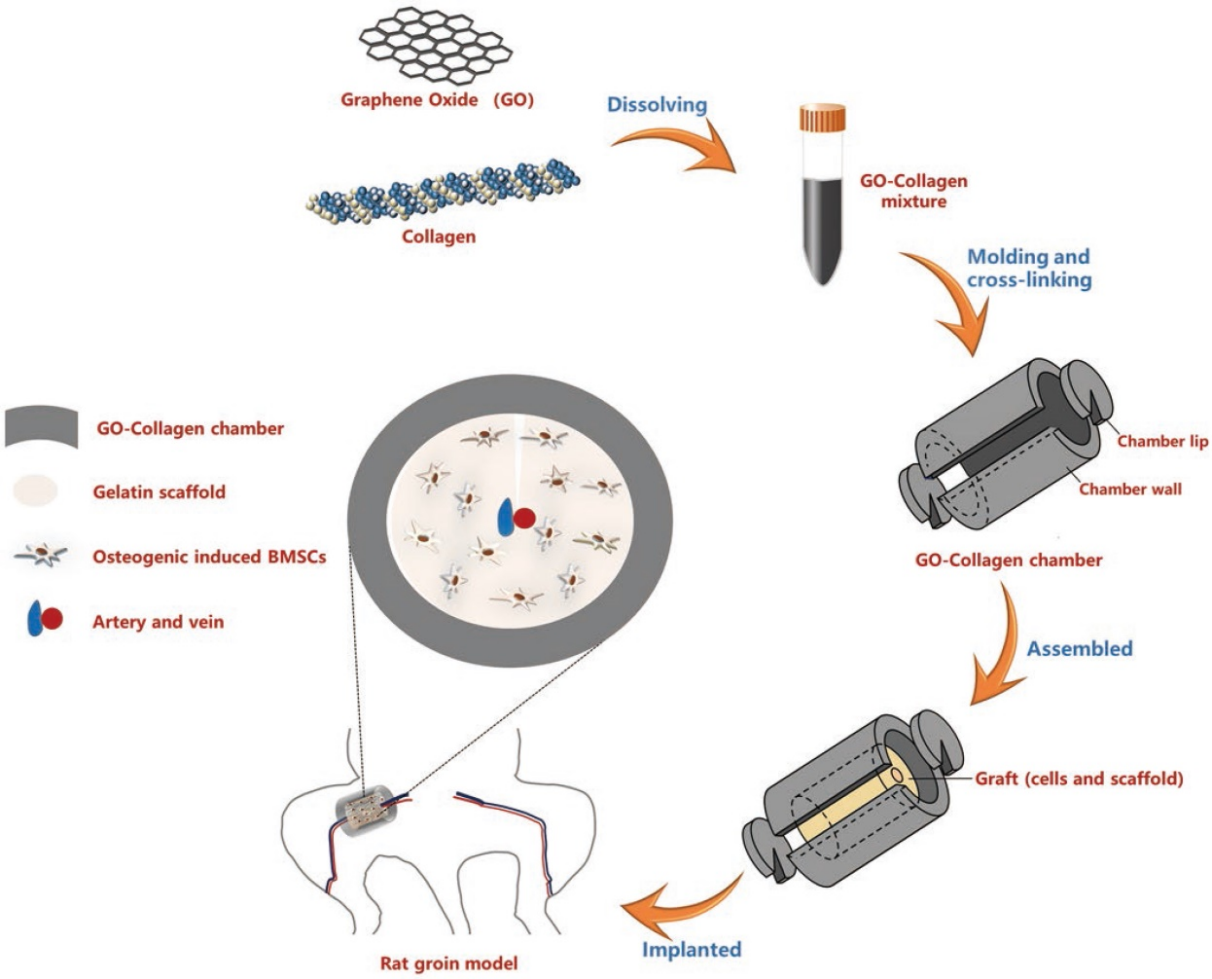
** Schematic illustration of the preparation and *in vivo* application of the GO-collagen tissue engineering chamber in a rat groin model.** Graphene oxide (GO) and collagen were dissolved, blended and injected into molds to obtain GO-collagen scaffolds with disc shape and hollow cylindrical shape. After the cross-linking process, GO-collagen scaffolds were fabricated to make a tissue engineering chamber. Then, the BMSCs-gelatin grafts were encased in the GO-collagen chamber and implanted into the rat groin area, with vessels traversing through the graft.

This study hypothesized that biocompatible GO-collagen is an ideal material for the construction of osteoinductive and anti-fibrosis effects tissue engineering chamber for bone tissue engineering. Herein, a hollow cylindrical GO-collagen tissue engineering chamber was constructed by injection of molding tool. The mechanical and biological properties of the materials were then characterized. Osteogenic induced bone mesenchymal stem cells (BMSCs)-gelatin grafts were embedded in the GO-collagen chamber with vessels traversing through the graft (**Figure 1**). Inflammatory responses were evaluated at different time points by measuring the expression of inflammatory cytokines and fibrous formation. Micro-computed tomography (CT) and histological examination were used in the detection of calcification and cell survival of osteogenic induced BMSCs-gelatin grafts. Also, the angiogenesis of the flow-through type vessels inside the chamber was detected.

## Methods

### Animals

All protocols used in the present study strictly adhered to the regulations and laws of China and conformed to the Standing Committee on Ethics in China (State Scientific and Technological Commission of China). Animal experiments were approved by the Department of Experimental Animals, Tongji Medical College, Huazhong University of Science & Technology (Wuhan, China), and conformed to the recommended guidelines.

### Construction and characterization of a GO-collagen chamber

GO aqueous dispersion solutions (0.1 wt%, 0.4 wt%, 1 wt%, Qingdao Huatai Tech. Co., Ltd, China) were prepared by sonication for 30 minutes with an ultrasonic processor (Branson, USA). Type I collagen solutions (2 wt%, 4 wt%, 6wt%, Chengdu Kele Science Technology. Co., Ltd, China) were prepared by dissolving and stirring collagen in 0.1% acetic acid on an ice bath for 30 minutes. The GO solutions and collagen solutions were mixed at a 1:1 volume ratio then sonicated for 30 minutes. Nine different concentrations of the mixture were injected into a cylindrical mold with an inner diameter of 6 mm and frozen at -20℃ overnight. Subsequently, the cylindrical molds lyophilized for 24 hours at -50℃ to form a porous structure. The crosslinking was achieved by immersing the materials in a solution (H_2_O: ethanol = 5: 95) containing 5wt% N- (3-Dimethylaminopropyl)- N-ethyl carbodiimide hydrochloride crystalline (EDC) (Sigma, Aldrich) and 2wt% N-hydroxysuccinimide (NHS) (Sigma, Aldrich) for 24 hours. After a thorough wash in distilled water, the materials were lyophilized at -50℃ to obtain a cross-linked GO-collagen composite construct. The collagen materials (1wt%, 2 wt%, 3wt%) were prepared using the same methods.

The 0.2wt%GO-2wt%collagen was selected for the construction of the tissue engineering chamber. GO-collagen constructs with disc shape (diameter: 7 mm; thickness: 1.5 mm) or hollow cylindrical shape (height: 10 mm; thickness: 1.5 mm; diameter: 10 mm ) were formed from pan-shaped molds and double-layer hollow cylindrical molds, respectively, using the same methods, and then fabricated by 5-0 surgical suture to make a GO-collagen chamber.

#### Water absorption

The water absorption capacity of the materials was calculated using the formula:


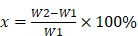


In which, W1 is the weight of GO-collagen materials and W2 is the weight of these materials filled with distilled water.

#### Mechanical property testing

In this test, the materials were molded into a cylinder with a height of 7 mm and a diameter of 6 mm. The stress-strain curve of these materials was tested using the All-Electric Dynamic Test Instrument (Electro Puls E1000, INSTRON, British).

#### MTT assay

BMSCs were seeded in GO-collagen and collagen materials to evaluate the biocompatibility of the constructs. Cell proliferation was measured by MTT assay as previously described 34.

#### Osteogenic induction

BMSCs were seeded in GO-collagen and collagen for 7 days, and cultured with osteogenic induction culture medium (Dulbecco's modified eagle medium containing 10% FBS, 0.1 μM dexamethasone, 10 mM β-glycerophosphate, and 50 μg/ml L-ascorbic acid) for two weeks to determine the osteogenic inductive ability of the materials.

#### Alizarin red staining

A subset of BMSCs-GO-collagen and BMSCs-collagen specimens were fixed, wrapped and cut into slices. Next, the slices were soaked in alizarin red S staining solution for 5 minutes and images were captured by phase-contrast microscopy (Ni-EH600L, Nikon, Tokyo, Japan).

#### Physical characteristics

The Dispersive Raman microscopy (FRA 106/s, Bruker, German) was used to examine the composition of the synthesized GO and GO-collagen materials. The functional groups of the collagen and GO-collagen materials were characterized by Fourier Transform Infrared Spectroscopy (FTIR, VERTEX 70, Bruker, German). Crystalline phases of GO and GO-collagen materials were evaluated by X-ray diffraction (XRD, Empyrean, Panalytical, Netherlands) with Cu Kα radiation (2θ range from 5° to 70°).

### Construction of the BMSCs-gelatin graft

#### Isolation of rat BMSC

Femurs and tibias were harvested from newborn Sprague Dawley (SD) rats (5 days old). Bone marrow cavities of the femurs and tibias were washed repeatedly with low glucose- DMEM medium supplemented with 10% FBS (DMEM, Hyclone, USA). Next, the washed-out contents were collected and seeded on culture dishes. The BMSCs were passaged every 3 days when the attached cells became confluent.

#### Preparation of gelatin scaffold

We prepared an aqueous solution of 6wt% gelatin (Sigma, Aldrich) in a water bath at 37℃. Next, 0.5wt% EDC and 0.2wt% NHS were added into the gelatin solution, and the mixture was injected into a tubulose mold (diameter: 7 mm) and frozen at -20℃ overnight followed by lyophilization for 24 hours at -50℃. Subsequently, a cylindrical gelatin scaffold was obtained (diameter: 7 mm; height: 7 mm) on which a groove was cut along its height.

#### Construction of the BMSCs-gelatin graft

The BMSCs were digested with 0.25% trypsin (Thermo Fisher Scientific) and resuspended at a cell concentration of 5×106/mL. Each gelatin scaffold was seeded on 60 μL cell suspension and cultured in 12-well plates. A total of 2.5 mL of complete medium (low glucose-DMEM containing 10% FBS) was added 1.5 hours after the adhesion process. After 7 days of cell culture, the complete medium was replaced by an osteogenic induction culture medium and the osteogenic differentiation was performed for 21 days. Finally, the tissue-engineered bone graft was obtained.

The BMSCs-gelatin tissue-engineered bone graft produced by the osteogenic induction process was fixed and dehydrated. The graft was sprayed with gold and the cells on gelatin scaffolds were examined with Scanning electron microscopy (SEM, S3400N, Hitachi, Tokyo, Japan). Alizarin red staining was performed as described before.

### *In vivo* implantation of the tissue engineering chamber in rat

Female adult SD rats (age: 3 months; weight: 250-300 g) obtained from the Laboratory Animal Center of Huazhong University of Science & Technology were used for this experiment. Rats were anesthetized through intraperitoneal injection of Ketamine (90 mg/kg) and Xylazine (9 mg/kg). After shaving and sterilizing the rat groin site, the skin was incised. Subsequently, the femoral artery and vein, which run below the skin, were identified. Next, the femoral vessels and iliac vessels were harvested from the surrounding tissue and put at the center of the implanted graft and the tissue engineering chamber. Animals were intramuscularly injected with penicillin for 3 days to prevent infection and sacrificed at different time-points to harvest specimens for further tests.

### Vascular perfusion

Two months after the implantation, the animals were anesthetized by intraperitoneal injection of Ketamine (90 mg/kg) and Xylazine (9 mg/kg) to perform vascular perfusion. A total of 9 rats were used for the procedure, and two samples were implanted per rat. The abdominal aorta and the inferior vena cava were exposed through a skin incision in the middle abdomen. Next, the two vessels were cannulated. The abdominal aorta was perfused with heparin saline to allow the blood would flow from inferior vena cava. After that, rats were sacrificed and the abdominal aorta was perfused with 200mL of 4% paraformaldehyde for fixation. It was then fixed with 200mL saline and then perfused with freshly prepared MICROFIL compounds (FlowTech, Inc. USA). The bodies of the rats were preserved in 4℃-overnight and the specimens were harvested the next day for further micro-CT and histological examinations.

### Micro-Computed Tomography

A total of 18 rats were used. The specimens were harvested after 1 or 3 months and evaluated using micro-CT (SKYSCAN 1176, BRUKER, Karlsruhe, Germany). The data from the scans were reconstructed using the VG studio (Volume Graphics GmbH, Heidelberg, Germany) to establish 3D models. The bone volume was calculated from quantitative morphometric analyses.

### Histology

#### Hematoxylin and Eosin staining

The specimens were fixed, wrapped, cut into sections, and then stained with Hematoxylin and Eosin (Sigma, St. Louis, USA). Images were captured by a phase-contrast microscope (Ni-EH600L, Nikon, Tokyo, Japan).

#### Von Kossa staining

The specimens were fixed and wrapped after which they were cut into thin sections. The sections were then stained with 2% silver nitrate solution, rinsed by sodium sulfate, and counterstained with fuchsine. The calcified area appeared dark in the sections. Images were captured by a phase-contrast microscope (Ni-EH600L, Nikon, Tokyo, Japan).

#### Osteocalcin staining

Slices were incubated with rabbit anti-rat osteocalcin primary antibody (Proteintech Group, Wuhan, China) and horseradish peroxidase (HRP)-conjugated anti-rat secondary antibody. Then, diaminobenzidine tetrahydrochloride was added for color development. Images were captured by a phase-contrast microscope (Ni-EH600L, Nikon, Tokyo, Japan).

#### CD68 staining

Sections were incubated with rabbit anti-rat CD68 primary antibody (Proteintech Group, Wuhan, China) and phycoerythrin-conjugated anti-rat secondary antibody. Images were captured by a laser scanning confocal microscope (Ni-E, Nikon, Tokyo, Japan).

#### CD31 staining

Sections were incubated with rabbit anti-rat CD31 primary antibody (Proteintech Group, Wuhan, China) and horseradish peroxidase (HRP)-conjugated anti-rat secondary antibody. Then, diaminobenzidine tetrahydrochloride was added for color development. Images were captured by phase-contrast microscopy (Ni-EH600L, Nikon, Tokyo, Japan).

### Real-time quantitative polymerase chain reaction analyses

The mRNA expression levels of IL-1β, IL-10, and TNF-α were detected and quantified by real-time qPCR. Total RNA was extracted from the specimens using Trizol Reagent kit (Thermos Fisher Scientific, USA). The concentration and quality of the RNA were determined using a spectrophotometer at 260/280 nm. Next, equal amounts of RNA from each sample were reverse-transcribed to cDNA. The cDNA was then used as a template for real-time qPCR in line with the manufacturer's instructions (Thermos Fisher Scientific, USA). Primers used for the RT qPCR were shown in **Table 1**.

### Assessment of the post-implantation fate of BMSCs

During the construction of the tissue-engineered bone graft, 5-Ethynyl-2'-deoxyuridine (EdU, Beyotime, Jiangsu, China) dissolved in the complete medium was added at a concentration of 10mM to label the BMSCs. Other procedures including the osteogenic induction and animal experiments were performed as previously described. The specimens were frozen-sliced for use in the click reaction to detect EdU-labeled cells. Images were captured by a phase-contrast microscope (Ni-EH600L, Nikon, Tokyo, Japan).

### Statistical analysis

All data are presented as mean ± standard deviation (SD). Student's t-test and one-way analysis of variance (ANOVA) were used for paired, and multiple comparisons, respectively, followed by post hoc contrasts by Student-Newman- Keuls test. P-values less than 0.05 were considered statistically significant.

## Results

### Physical properties of GO-collagen

Different concentrations of GO-collagen were prepared by cylindrical molding. GO-collagen materials exhibited optimal mechanical properties at 2% collagen concentration **(Figure S1A)**. The concentration of collagen based on the water absorption capacity of GO-collagen **(Figure S1B)**. Collagen (2%) with different concentrations of GO was selected to detect the elasticity and plasticity. Dynamic compression test was used to get the strain-stress curves of different materials with 2%wt collagen. In the strain-stress curves (at the same compressive strain), higher stress reflected better rigidity of the material. According to the results, a higher concentration of GO enhanced the mechanical property of collagen. The slope of the strain-stress curves represented the elasticity modulus of the materials at the plastic state (typically, higher elasticity modulus means the materials are more brittle). The plasticity of the materials declined with an increase in GO concentration. The strain-stress curves showed that the elastic modulus of 0.2wt%wt GO- 2wt%collagen was 0.7465MPa, which had better plasticity than 0.5wt%GO-2wt%collagen with an elasticity modulus of 2.269PMa **(Figure S1C)**. MTT assay showed that the concentration of GO did not affect cell proliferation in the composite materials **(Figure S1D)**. The 0.2wt%GO-2wt% collagen concentration was selected for use in the construction of the tissue engineering chamber. The results showed that BMSCs grew well in GO-collagen, indicating that the material was biocompatible **(Figure S1E)**. The BMSCs were seeded on GO-collagen and collagen scaffolds. The Von Kossa staining showed that more BMSCs osteogenic differentiated in GO-collagen scaffold **(Figure S1F)** than in the collagen material **(Figure S1G)**, which suggested that GO accelerated the osteogenic process.

Hematoxylin-Eosin staining was performed to observe the *in vivo* degradation of GO-collagen at different time points. Within 3 days, inflammatory cells had accumulated on the surface of GO-collagen **(Figure S2A),** and within 10 days, macrophages migrated and gathered to phagocytize the GO-collagen **(Figure S2B)**. A thin layer of the fibrous capsule was observed in 20 days **(Figure S2C)**. The GO-collagen materials were continuously degraded by the macrophages and the fibrous capsule and later filled with black macrophages **(Figure S2D)**.

### Characteristics of the GO-collagen chamber and BMSCs-gelatin grafts

**Figure 2:**
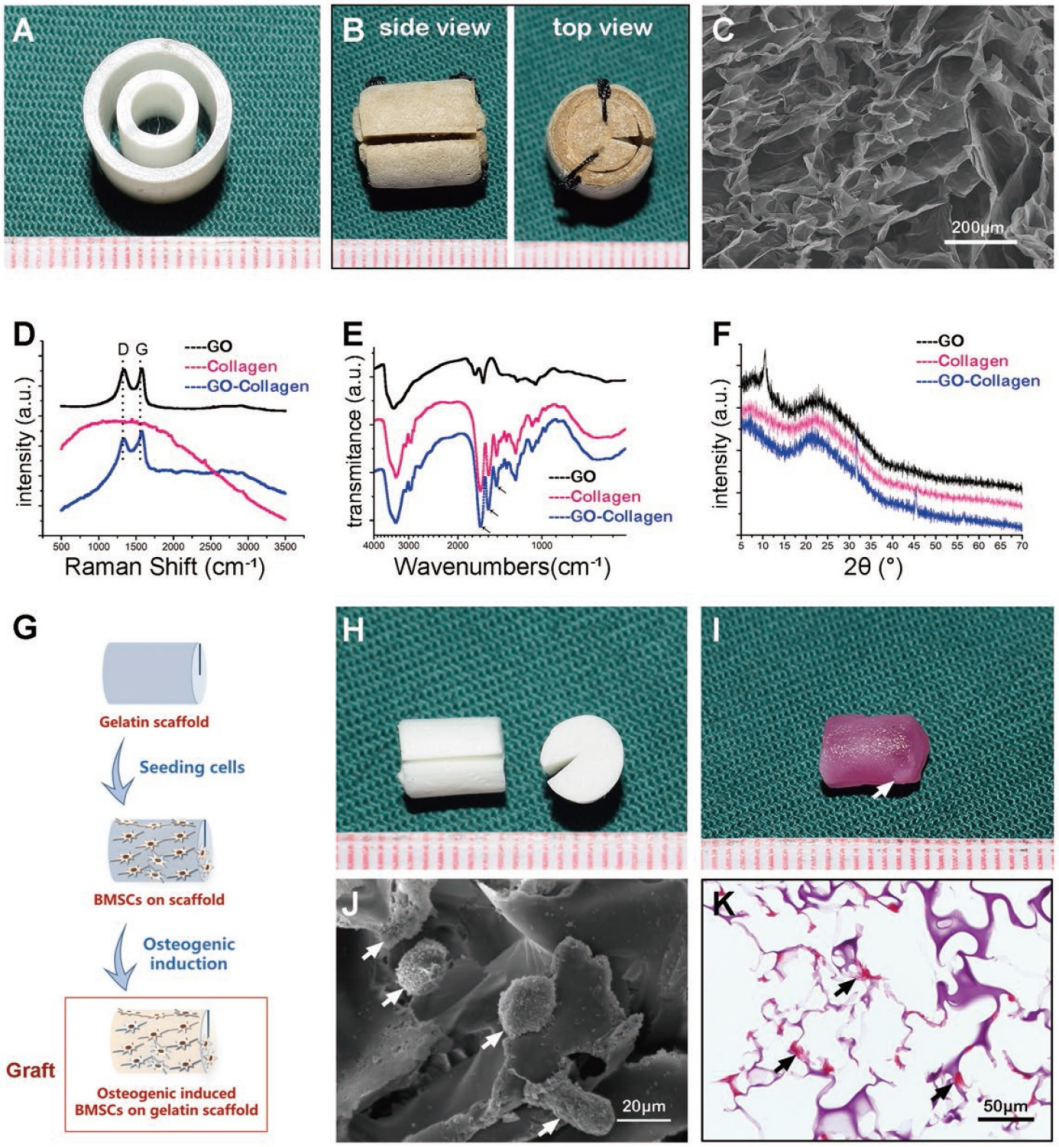
** Characterization of GO-collagen chamber and BMSCs-gelatin grafts.** GO-collagen chamber **(B)** was formed from the hollow cylindrical mold **(A)** and characterized by scanning electron microscope (SEM) **(C)**, Raman spectroscopy **(D)**, Fourier infrared spectrometer **(E)** [Black arrow: a characteristic peak of collagen], and X-ray diffractometer **(F)**. Rat bone marrow mesenchymal stem cells (BMSCs) were seeded onto gelatin scaffolds. The osteogenic induction process was run for 21 days to construct a tissue-engineered bone graft **(G-I, I,)** [white arrow: calcified area in tissue-engineered bone graft]. SEM showing the cells on gelatin scaffold **(J)** [white arrow: BMSCs]. Alizarin red staining indicated the osteogenic differentiation of BMSCs **(K)** [black arrow, osteogenic differentiated BMSCs].

A 3D printer was used to construct hollow cylindrical polydimethylsiloxane molds **(Figure 2A)**. The GO-collagen mixture was injected into the molds to obtain GO-collagen with a disc shape and a hollow cylindrical shape. After vacuum drying, cross-linking, and assembling processes, the GO-collagen tissue engineering chamber was constructed **(Figure 2B)**. Scanning electron microscopy image of GO-collagen showed the porous structure of this material **(Figure 2C)**. Raman microscopy was used to determine the GO, collagen and GO-collagen composition. And typical D bond and G bond of GO were found in the GO-collagen material **(Figure 2D)**. Fourier Transform Infrared Spectroscopy was used to show the characteristic peaks of the amide bond in collagen and GO-collagen **(Figure 2E)**. X-ray diffraction was used to detect the crystalline phases of the materials. When 2θ was 11°, a diffraction peak appeared in GO, but disappeared in GO-collagen material, indicating that GO had been reduced and dispersed into a single layer during preparation **(Figure 2F)**.

BMSCs-gelatin tissue-engineered bone grafts were built by BMSCs and gelatin scaffolds **(Figure 2G)**. The gelatin scaffolds were designed with a groove to embed the vessels **(Figure 2H)**. The BMSCs-gelatin tissue-engineered bone grafts were obtained after cell seeding and osteogenic induction **(Figure 2I)**. Scanning electron microscopy images showed the cells on the gelatin scaffold **(Figure 2J),** whereas Alizarin red staining showed the osteogenic differentiated cells **(Figure 2K)**.

### Constructed tissue engineering chamber model in rat groin

**Figure 3:**
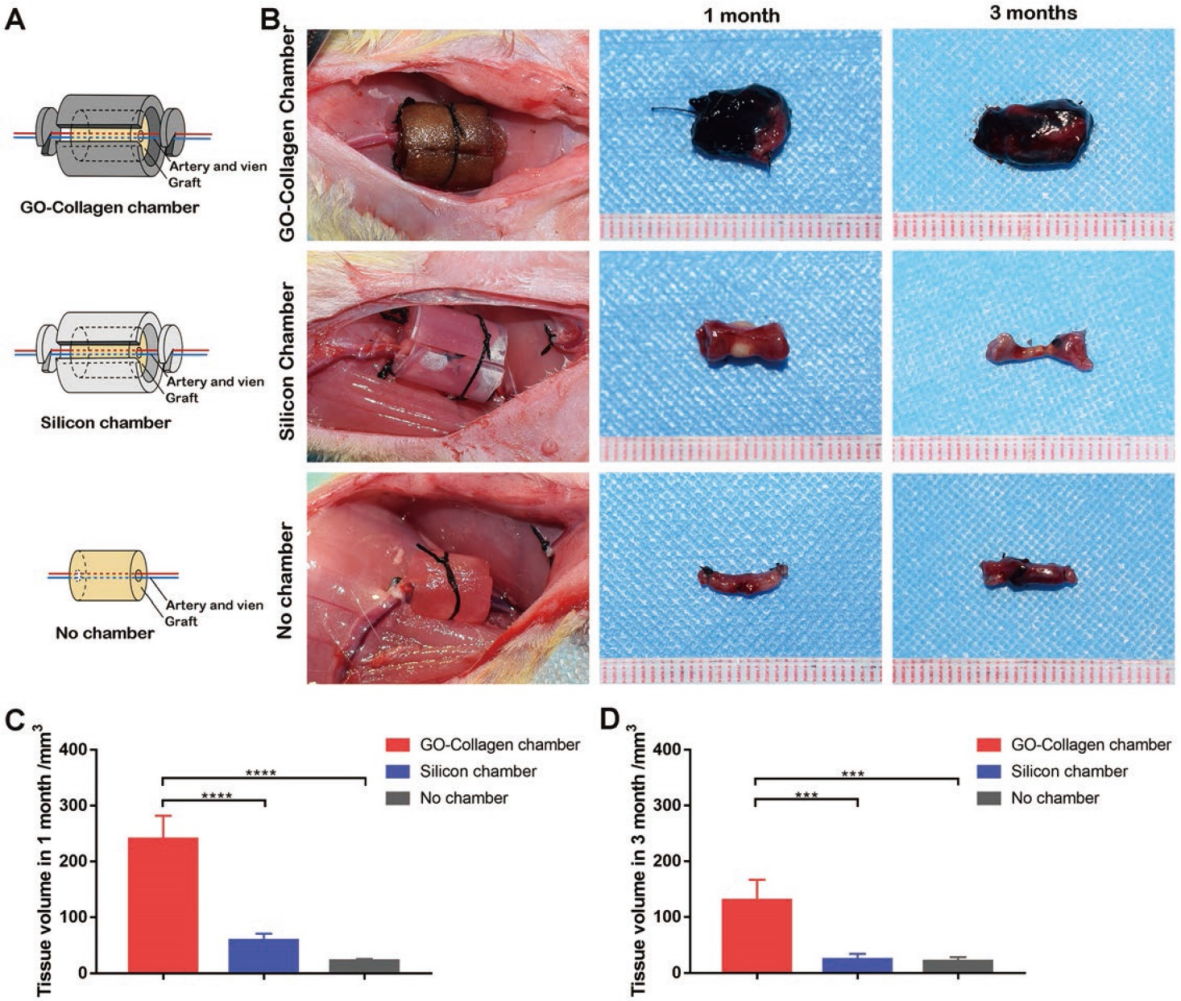
** GO-Collagen chamber in rat groin model.** BMSCs-gelatin grafts were encased in different chambers (GO-collagen chamber group, silicone chamber group and no chamber group). Then, the chambers were implanted and fixed in the rat groin area with vessels traversing through the grafts **(A)**. Macroscopic views of the implants at different time-points (1 month, 3 months) showed that the GO-collagen group had the largest tissue volume among the three groups **(B)**, as further confirmed by quantitative analysis **(C-D)**.

The BMSCs-gelatin tissue-engineered bone grafts were encased into GO-collagen and silicone chambers and implanted into the rat groin area, with vessels traversing through the center of the grafts** (Figure 3A)**. The grafts were taken out and the animals sacrificed after one or three months after implantation **(Figure 3B)**. Representative macroscopic views indicated that the grafts were highly degraded in the silicone chamber and no chamber groups; however, the grafts sustained proper morphology and tissue volume in the GO-collagen chamber group. The tissue volume of each group was measured according to Archimede's principle, and the results showed that the tissue volume was more than three folds higher (p<0.05) in the GO-collagen chamber group than in the other two groups **(Figure 3C-D)**.

### Osteogenesis in tissue engineering chamber

**Figure 4:**
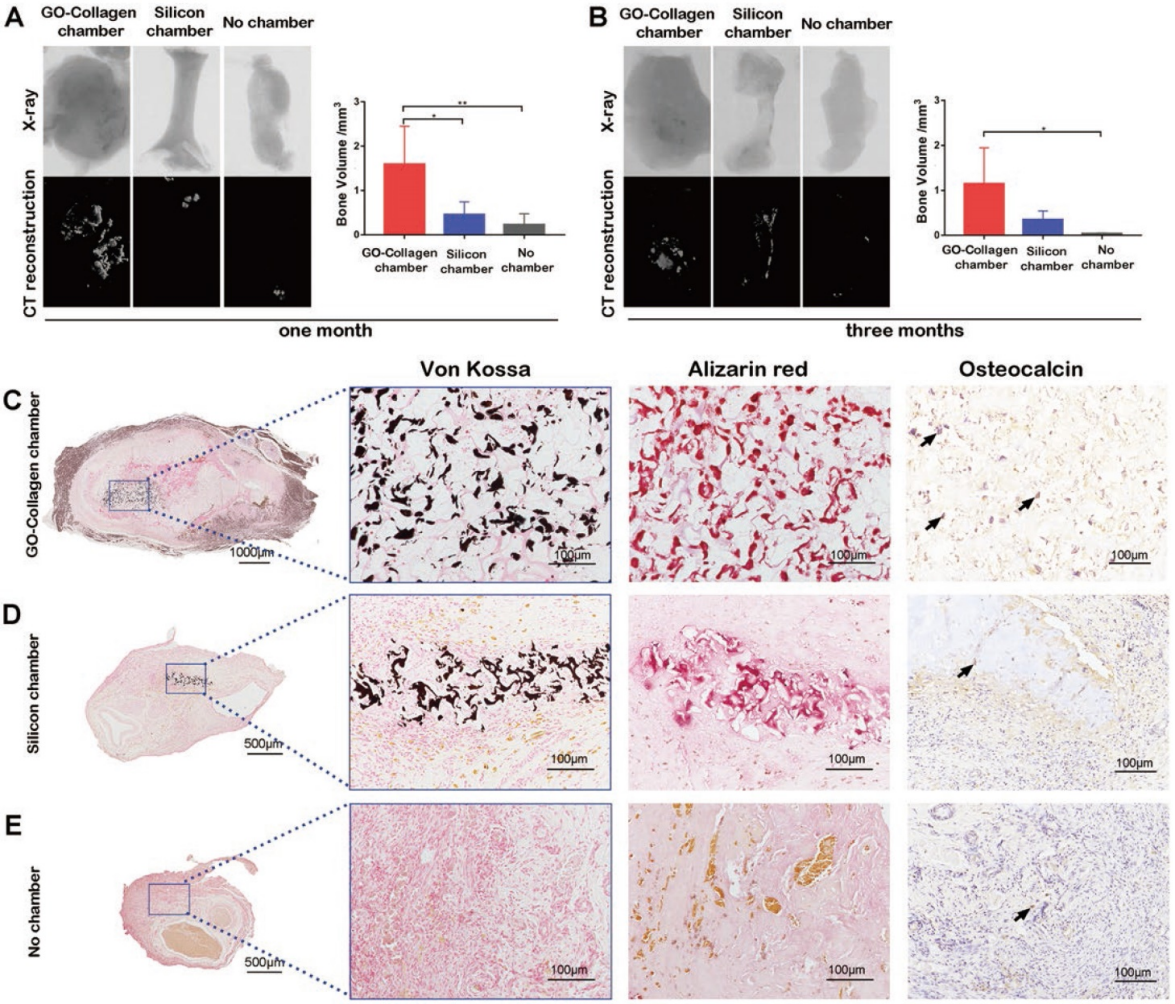
** The osteogenic performance of BMSCs-gelatin graft in the GO-collagen chamber.** Representative X-ray and CT reconstruction images of the implants show a high number of mineralized tissues in the GO-collagen chamber than other groups **(A-B)**. Quantitative analysis of micro-CT shows larger bone volume in the GO-collagen chamber group than the other two groups both 1 month **(A)** and 3 months **(B)** after implantation. Von Kossa, Alizarin red and osteocalcin were performed at 1 month after implantation to evaluate the ectopic bone formation **(C-E)**. Remarkably, more mineralized tissue and osteoblasts were found in the GO-collagen chamber than in the silicone chamber in Von Kossa, Alizarin Red and osteocalcin analysis. In contrast, there were few bony tissues or osteocalcin positive cells in the entire area of the implants in the no chamber group **(C-E)** [black arrow: osteocalcin^+^ cells].

Micro-CT reconstruction and X-ray scanning were performed to detect the osteogenesis in the tissue engineering chamber one or three months after the implantation. Representative images and quantitative analysis of bone volume in the different groups showed that there was increased calcification tissue formed in the GO-collagen chamber than in the other two groups **(Figure 4A-B)** (p<0.05). The Von Kossa and Alizarin red stainings of specimens in these groups confirmed that the high-density tissue in CT images were calcific areas of the tissue-engineered bone graft **(Figure 4C-E)**. The results showed that in the GO-collagen chamber, the BMSCs-gelatin grafts survived better with larger volume and calcific area.

### Inflammatory reactions in the tissue engineering chamber

**Figure 5:**
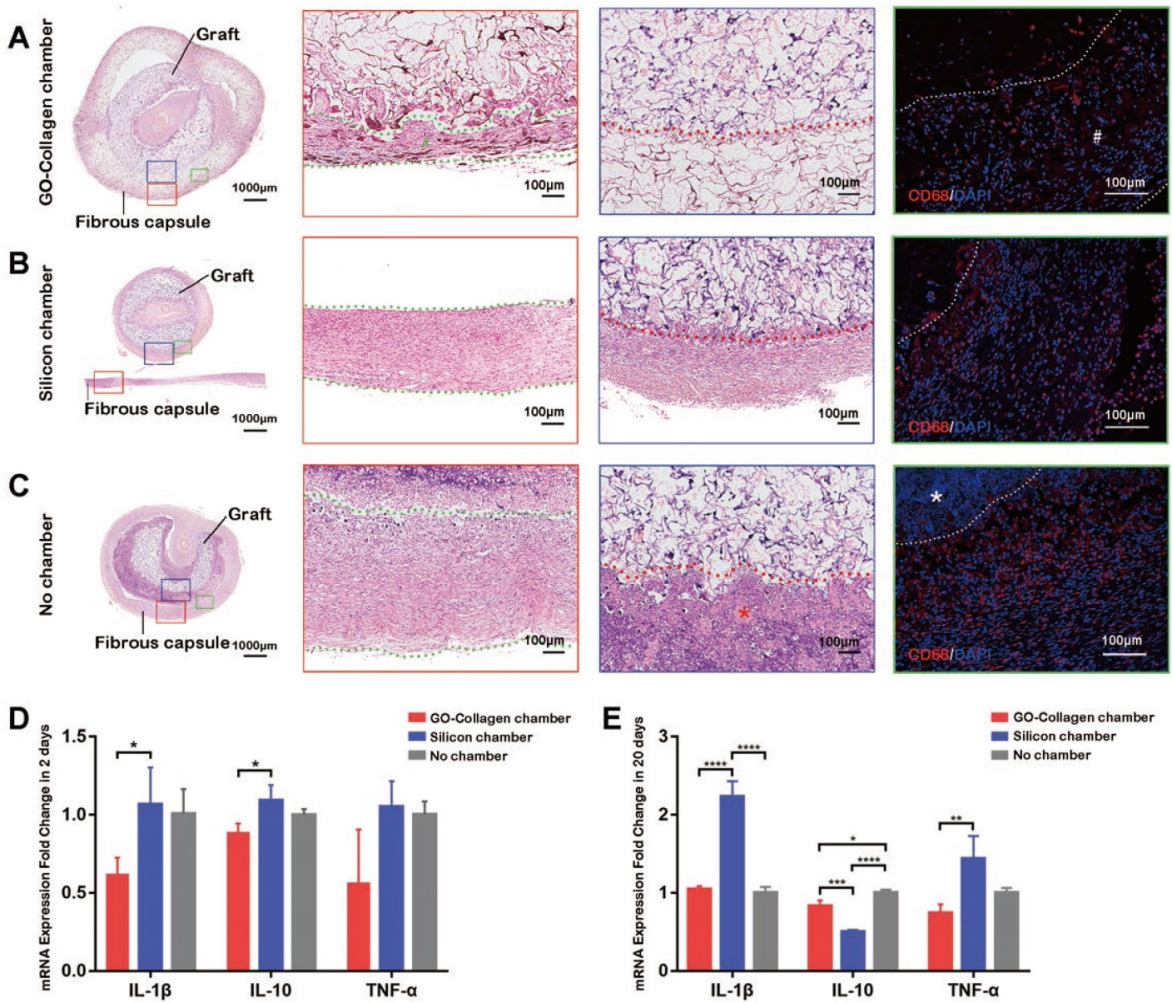
** Inflammatory response to GO-Collagen chamber in rat groin model.** Hematoxylin-Eosin staining was performed to detect the formation of a fibrous capsule in different groups** (A-C)** [red boxes, between green dotted line]. CD 68 immunofluorescence showed that few macrophages assembled in the region of grafts in the GO-collagen chamber group, indicating high biocompatibility of the GO-collagen chamber (**A-C**, green boxes). Analysis of inflammatory-related gene expression (IL2, IL10, TNF-α) revealed that the GO-collagen chamber caused milder inflammatory reactions than the other two groups at both early (2 days) and later (20 days) stages **(D-E)**.

Hematoxylin-Eosin staining and CD68 immunofluorescence staining (black and white scale bars: 100μm) on the tissue-engineered bone grafts were done twenty days after the implantation **(Figure 5A-C)**. A thin layer of the fibrous capsule formed around the GO-collagen chamber **(Figure 5A, red box, #)**. The fibrous capsule in the silicone chamber and no chamber groups were denser than those in the GO-collagen **(Figure 5B-C, red box)**. The fibrous capsule and the graft inside the chamber were fixed and sliced separately because the silicone chamber could not be sliced together with the tissue **(Figure 5B)**. The GO-collagen chamber wrapped the grafts and protected them from the inflammatory cells **(Figure 5A, blue box)**. Interstitial fluid infiltrated into the silicone chamber causing inflammatory cells to aggregate around the graft **(Figure 5B, blue box)**. In the no chamber group, there was a layer of necrotic cells under the fibrous capsule (Figure 5C, *). The CD68 immunofluorescence staining indicated that there was less macrophage infiltration in the GO-collagen chamber than in the other two groups **(Figure 5A-C, green boxes)**.

Real-time qPCR was used to detect and quantify the mRNA expression level of three inflammatory genes (IL-1β, IL-10, and TNF-α) 2 and 20 days after implantation. The GO-collagen chamber group exhibited lower expressions of IL-1β and IL-10 compared to the silicone chamber group 2 days post-surgery **(Figure 5D)** [p<0.05]. Also, the GO-collagen chamber group showed lower expressions of IL-1β and TNF-α and higher expressions of IL-10 than silicone chamber group 20 days post-surgery **(Figure 5E)** [p<0.05].

### Neovascularization in different groups

**Figure 6:**
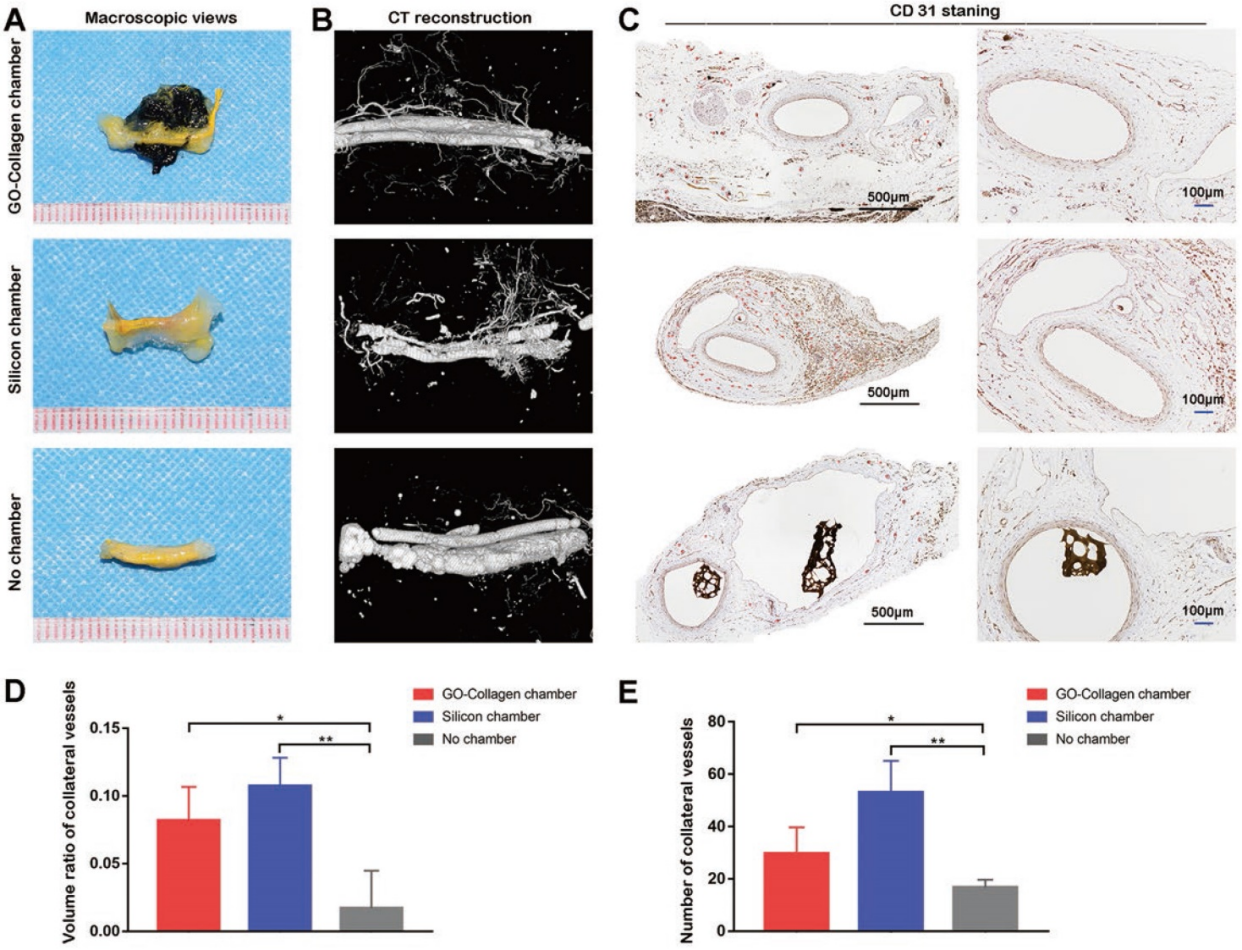
** The angiogenesis performance of the wrapped BMSC-gelatin grafts at 2 months post-implantation.** Vascular perfusion and CT reconstruction were performed to evaluate the neovascularization state in the grafts. Angiogenic sprouting occurred in the GO-collagen chamber and the silicone chamber groups **(A-B)**. CD31 immunohistochemical staining also demonstrated more neo-vessels formation (red *) in the GO-collagen chamber and silicone chamber groups than in no chamber group **(C)**. Quantitative analysis confirmed that both the volume ratio of neo-vessels and the number of neo-vessels were higher in the GO-collagen chamber and silicone chamber groups than in no chamber group **(D-E)**.

After the vascular perfusion process, vessels in the grafts were filled with yellow contrast agent. As revealed by the macroscopic images, many collateral vessels grew from the center in the GO-collagen chamber and silicone chamber groups, but only a few collateral vessels were observed in the no chamber group **(Figure 6A)**. The results of micro-CT reconstruction images were consistent with those of the macroscopic images **(Figure 6B)**. The immunohistochemical staining of CD31 also confirmed the imaging results **(Figure 6C)**. The micro-CT data (n=5) was used to calculate the volume ratio of collateral vessels **(Figure 6D)**. The CD31 staining sections (n=5) were used to count the number of collateral vessels under light microscopy **(Figure 6E)**. The two histograms **(Figure 6D-E)** confirmed the angiogenesis effect in both the GO-collagen chamber and the silicone chamber groups.

### Post implantation fate of BMSCs in the tissue engineering chamber

**Figure 7:**
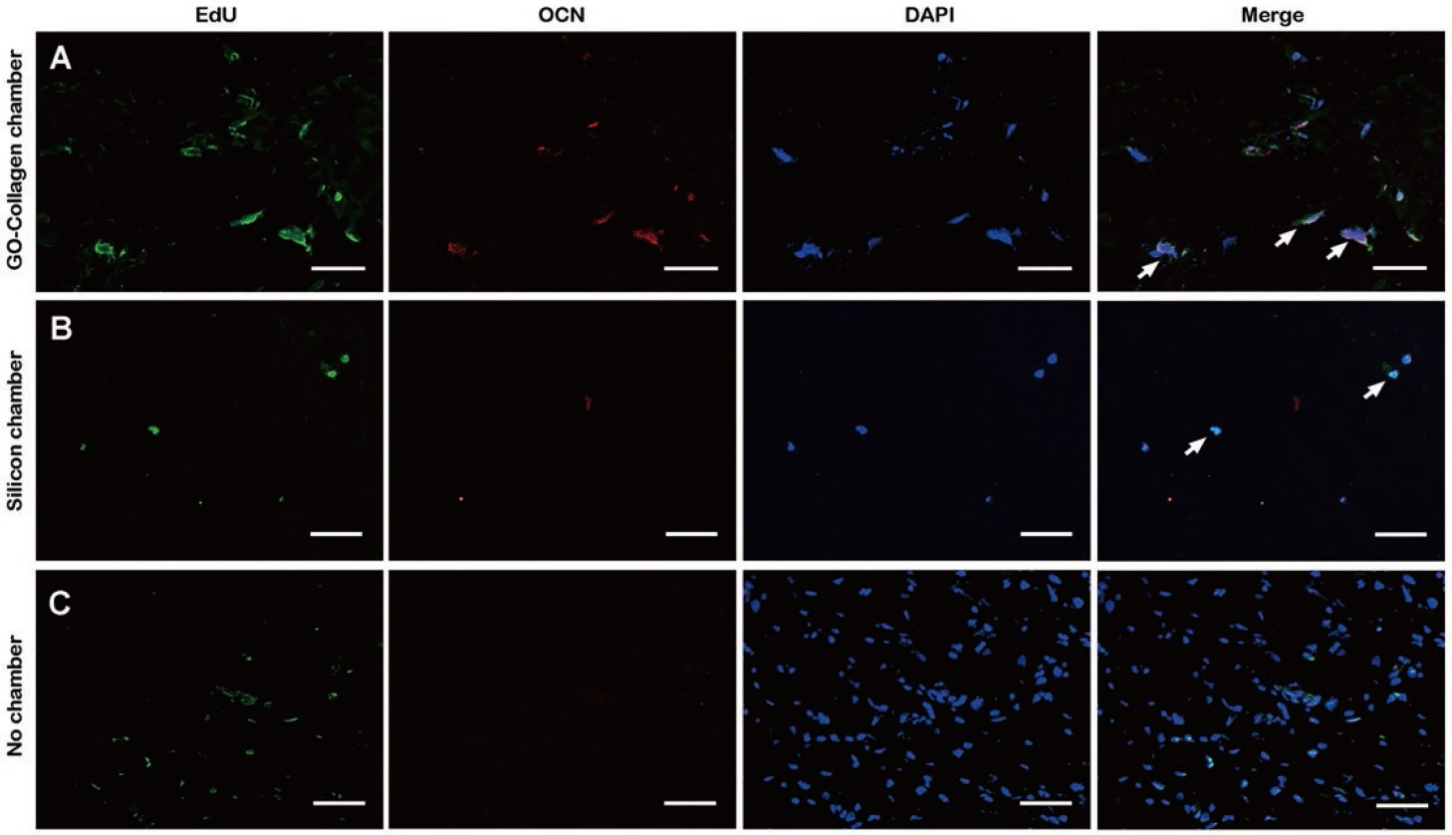
** Post-implantation fate of osteogenic induced BMSCs in the tissue engineering chamber.** EdU-labeled BMSCs were seeded on gelatin scaffolds and induced to undergo osteogenic differentiation. One month after implantation in the rat groin, immunofluorescence tests showed that a higher number of BMSCs were differentiated into pre-osteoblasts or osteoblasts 1 month after implantation in GO-collagen group compared to the other groups **(A-C)** [scale bar: 50μm].

The BMSCs in the tissue-engineered bone grafts were labeled with 5-ethynyl-2´-deoxyuridine (EdU) before implantation. The animals were sacrificed, and the grafts were sliced up for fluorescence staining to locate EdU labeled cells one month after the implantation. In the GO-collagen chamber group, BMSCs were more, and some of them had differentiated to express osteocalcin **(Figure 7A)**. In the silicone chamber group, a large proportion of the graft had degraded; therefore, only a few labeled BMSCs were observed in the remaining gelatin scaffold **(Figure 7B)**. In the no chamber group, BMSCs-gelatin tissue-engineered bone graft was quickly degraded and replaced by fibrous tissue with some of the cells in fibrous tissue emitting weak green fluorescence **(Figure 7C)**.

## Discussion

In this study, the GO-collagen tissue engineering chamber model was constructed to provide a suitable microenvironment with osteoinductive and anti-fibrosis dual effect, which promoted bone regeneration *in vivo*. Compared to silicone group and no chamber group, the semi-biodegradable GO-collagen group displayed the following advantages: (1) better biocompatibility with the ability to reduce fibrous formation and protect the transplanted tissues from the infiltration of macrophages; (2) preserve the transplanted constructs inside the chamber with the largest calcific area and more survived BMSCs.

The mechanical strength of GO-collagen improved with an increased concentration of GO **(Figure S 1C)**. This increase was associated with the high degree of crosslinking which produced a higher degree of geometric constraint on the mobility of the polymer chains of the scaffold 27 . According to the strain-stress curves, 0.2wt%GO-2%collagen exhibited good mechanical strength and plasticity and could be molded into different shapes. Moreover, the hollow cylindrical GO-collagen tissue engineering chamber was designed based on the structure of the traditional silicone tissue engineering chamber **(Figure 2B)**.

The tissue volume **(Figure 3)** and the calcified area **(Figure 4)** were significantly higher in the GO-collagen chamber group than in the other two groups. And could be attributed to the osteoinductive effect of GO and collagen. As the main structural protein of bone matrix, collagen accelerates the differentiation of bone progenitor cells and subsequently elicits cell growth and mineral production 35. Studies have shown that GO has osteogenic inductive potential 36,37. Also, our previous studies indicated that GO-collagen scaffolds could accelerate the repair of bone defect *in vivo* 25,38. Graphene oxide stimulates early osteogenic gene expression and calcium deposition of BMSCs through the upregulation of oncostatin M (OSM) and bone morphogenetic protein-2 (BMP2) 33. The activation of the OSM signaling pathway promotes osteogenesis by activating signal transducers and activators of transcription 3 (STAT3) and BMP2 (a growth factor which promotes osteogenesis) 39.

The formation of a thick and dense fibrous capsule around the implants can prevent intimate integration of the implant with the surrounding tissues and block nutrient exchange leading to cell death and transplantation failure 40. Also, the implanted cells may die as a result of foreign body reaction. And the dead cells may be cleared by the macrophages, thus reducing the volume of the grafts 41. Therefore, reducing the foreign body reaction and improving the biocompatibility of the implant materials could provide better survival conditions for the transplant 42,43 . In this study, histological imaging showed that the fibrous capsule was thinner in the GO-collagen chamber group than in the other two groups **(Figure 5A-C)**. These findings were consistent with our hypothesis and supported the anti-fibrosis effect of GO-collagen. Fewer macrophages aggregated around the tissue-engineered bone graft in the GO-collagen chamber group **(Figure 5A-C)**, which suggested that the GO-collagen had better tissue compatibility 44,45. Inflammatory cytokines, including interleukin-1β (IL-1β) and tumor necrosis factor-α (TNF-α), can induce the recruitment and activation of leukocytes, stimulate ROS production and additional cytokines that can damage and destroy the transplanted cells 46. Interleukin-10 (IL-10) is a general suppressive cytokine which represses proinflammatory responses and limits unnecessary tissue disruptions caused by inflammation 47. The GO-collagen chamber group showed lower expressions of IL-1β and TNF-α and higher expressions of IL-10 than the silicone chamber group, 20 days after implantation **(Figure 5E)**. These findings revealed the biocompatibility of the GO-collagen chamber and were consistent with the results of many studies that have confirmed the long-term compatibility of GO and GO degradation products *in vitro* and *ex vivo*. The toxicity of GO to intraperitoneal injected and treated animals is insignificant 48. Graphene oxide nanoparticles are eliminated from the body through the hepatobiliary route after intravenous injection 49, suggesting that GO-collagen might be a suitable material for biomedical applications *in vivo*.

Vascularization remains one of the main obstacles in the clinical application of tissue engineering (TE); thus several attempts have been made in the development of vascularized graft tissues 1,50. Many studies have shown that tissue engineering chamber model provides a pre-vascularized microenvironment for the transplanted construct 7,51. In tissue engineering chamber, pre-embedded vessels form membrane blebs that lead to vascular sprouts and the neovessels simultaneously assemble into a robust capillary bed to support the survival of a variety of constructs 52. This model has been successfully used to construct various tissues and cells 9,11,53 . Morrison successfully created large adipose tissue constructs in patients using the tissue engineering chamber and this pioneering work confirmed that the TEC model could be successfully used in humans 18,54. Arteriovenous loop (AVL) chamber and flow through chamber are the two main ways used to achieve vascularization 7,55. In the present study, a flow-through chamber model was adopted because it does not require harvesting of vein grafts and vascular anastomosis which allows the vessels inside the chamber to be left in continuity. Angiogenesis was observed in both the GO-collagen chamber and the silicone chamber groups **(Figure 6A-C)**. This was an indication that the GO-collagen tissue engineering chamber provided a vascularized microenvironment similar to the one provided by the traditional tissue engineering chamber. The chamber wall was presumed to have acted as a shell, providing a stable microenvironment and cushioning the pressure from surrounding tissues 9. In the no chamber group, both the tissue-engineered bone graft and the vessels suffered high pressure from the surrounding fibrous capsule and muscles due to limited support from the chamber. No significant difference in angiogenesis was observed between the GO-collagen chamber and silicone chamber groups (**Figure 6D-E**), which suggested that the composition of the chambers lacked growth factors that can promote angiogenesis 56. Therefore, further studies should focus on finding biomaterials that can promote angiogenesis for the construction of tissue engineering chamber. Alternatively, crosslink vascular growth factors in the GO-collagen chamber can be adopted in subsequent studies.

Many attempts have been made to improve cell survival after *in vivo* implantation. For instance, osteogenic biomaterials have been extensively investigated for *in vivo* bone regeneration 12,13. It has been found that bioactive nanocomposite hydrogels promote *in situ* bone regeneration by acting as carriers that release bioactive ions and small molecule drugs that enhance the survival and differentiation of stem cells 12. In most cases, the implanted cells die as a result of harsh microenvironment conditions such as anoikis and inflammation 57. Therefore, therapeutic outcomes of stem cell transplantation therapy are highly dependent on the fate of the implanted cells. So far, tissue engineering chamber has been reported to support the engraftment and survival of transplanted cells by interacting with the host capillaries which supply oxygen, nutrient, and remove waste materials 7,9,18. In this study, confocal images indicated that a higher number of BMSCs survived and differentiated in the GO-collagen tissue engineering chamber one month after the surgery compared to the control group **(Figure 7A)**. The green fluorescence of EdU was distributed in the fibrous capsule area in the no chamber group **(Figure 7C)**, which could have been as a result of the degradation of the BMSC-gelatin grafts following the fibrous capsule formation and macrophage aggregation. The neovessels in the GO-collagen chamber supplied nutrients and oxygen, thus promoting the survival of more cells in the GO-collagen chamber than in no chamber group 51. Also, the GO-collagen promoted the osteogenic differentiation of BMSCs and this demonstrated that the GO-collagen tissue engineering chamber had osteogenic inductive and anti-fibrosis dual effects in bone regeneration.

In summary, a suitable *in vivo* microenvironment for transplanted tissue should be well vascularized to provide a sufficient supply of nutrients. Besides, the inflammatory reaction after implantation should be mild 4. Biomaterials with biological activity play an essential role in the survival and differentiation of transplanted tissues 5,6. In this study, a biocompatible vascularized GO-collagen chamber model for bone regeneration was developed. The tissue engineering chamber acted as a shell to protect the inside tissue. The chamber wall with the GO component helped with the osteogenesis of BMSC and reduced the inflammatory reaction. This novel strategy of constructing a tissue engineering chamber with high osteogenic activity for bone regeneration can be applied in other fields. For instance, customized chambers from different biomaterials can be used for *in vivo* culture of other cell types and constructs.

## Conclusions

In conclusion, this study created a biocompatible vascularized GO-collagen chamber model, which enhanced the osteogenesis process of BMSC and decreased macrophage chemotaxis and the formation of a fibrous capsule leading to improved bone regeneration. In the GO-collagen group, there was significant bone mineralization, milder inflammatory reactions and more surviving cells than in the silicone and no chamber groups. This study shows that highly biocompatible tissue engineering chambers can offer a customized microenvironment, a property that is vital in regenerative medicine research.

## Supplementary Material

Supplementary figures.

## Figures and Tables

**Table 1:** Primers used for the RT qPCR.

Gene	Primer sequence
R-GAPDH-S	CTGGAGAAACCTGCCAAGTATG
R-GAPDH-A	GGTGGAAGAATGGGAGTTGCT
R-TNFα(RZ)-S	TGATCCGAGATGTGGAACTGG
R-TNFα(RZ)-A	CTCCTCCGCTTGGTGGTTT
R-IL10-S	CACTGCTATGTTGCCTGCTCTT
R-IL10-A	GTCTGGCTGACTGGGAAGTGG
R-IL1b-S	TGACCTGTTCTTTGAGGCTGAC
R-IL1b-A	CATCATCCCACGAGTCACAGAG

## Abbreviations

GO: graphene oxide
BMSC: bone mesenchymal stem cells
CT: Micro-computed tomography
EDC: N- (3-Dimethylaminopropyl)- N-ethyl carbodiimide hydrochloride crystalline
NHS: N-hydroxysuccinimide
MTT: 3-(4,5-dimethylthiazolyl-2)-2,5-diphenyltetrazolium bromide
DMEM: Dulbecco's Modified Eagle's Medium
FBS: fetal bovine serum
FTIR: Fourier Transform Infrared Spectroscopy
XRD: X-ray diffraction
SEM: Scanning electron microscopy
HRP: horseradish peroxidase
HE: Hematoxylin-Eosin
mL: Milliliter
PCR: polymerization chain reaction
EdU: 5-Ethynyl-2'-deoxyuridine
