# Supplementary material for: A biocompatible vascularized graphene oxide (GO)-collagen chamber with osteoinductive and anti-fibrosis effects promotes bone regeneration in vivo
Source: Theranostics. 2020 Feb 3;10(6):2759–72. doi: 10.7150/thno.42006 (PMC7052891; doi:10.7150/thno.42006)
Supplement: Supplementary file 1 — Supplementary figures. [file thnov10p2759s1.pdf]

Supplementary material

A biocompatible vascularized graphene oxide (GO)-collagen chamber with osteoinductive and anti-fibrosis effects promotes bone regeneration in vivo

Huimin Fang#, Chao Luo# , Shaokai Liu, Muran Zhou, Yuyang Zeng, Jinfei Hou, Lifeng Chen, Shan Mou, Jiaming Sun*, Zhenxing Wang*

Department of Plastic Surgery, Union Hospital, Tongji Medical College, Huazhong University of Science and Technology, Wuhan 430022, China.

# These authors contributed equally to this work.

* Corresponding authors

Corresponding authors' e-mail addresses:

*Zhenxing Wang: wangzhenxing@hust.edu.cn

*Jiaming Sun: sunjm1592@sina.com

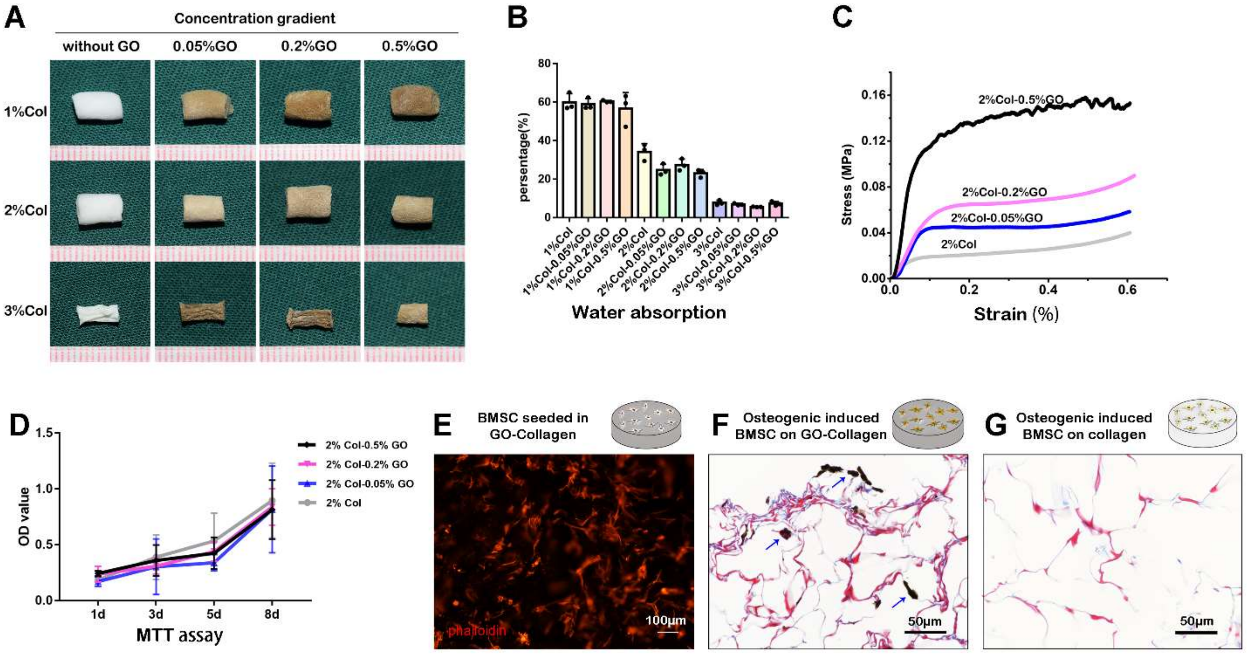

Figure S 1: GO-collagen displayed good mechanical properties and biocompatibility at optimal concentration of GO and collagen.

Different concentrations of GO and collagen composite materials were synthesized, and materials with 2% collagen displayed better plasticity (A). Water absorption and dynamic compression tests showed that the concentration of collagen and GO influenced the water absorption and stiffness, respectively (B-C). Rat BMSC were seeded on GO-collagen, and the biocompatibility of these materials were tested by MTT assay (D) and cytoskeleton staining (E). The Von Kossa staining showed that more BMSCs osteogenic differentiated in GO-collagen scaffold (F) than in the collagen material (G).

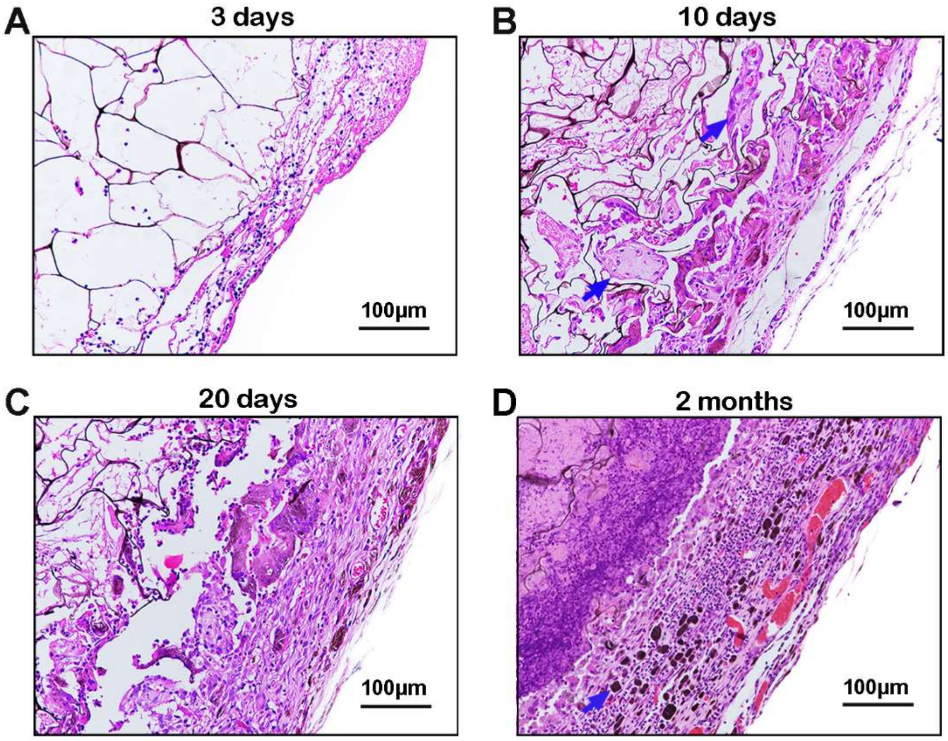

Figure S 2: Degradation of GO-collagen chamber in vivo.

Hematoxylin-Eosin staining was performed at different time-points and the results indicated that the GO-collagen was continuously degraded by macrophages (blue arrow) (A-D).

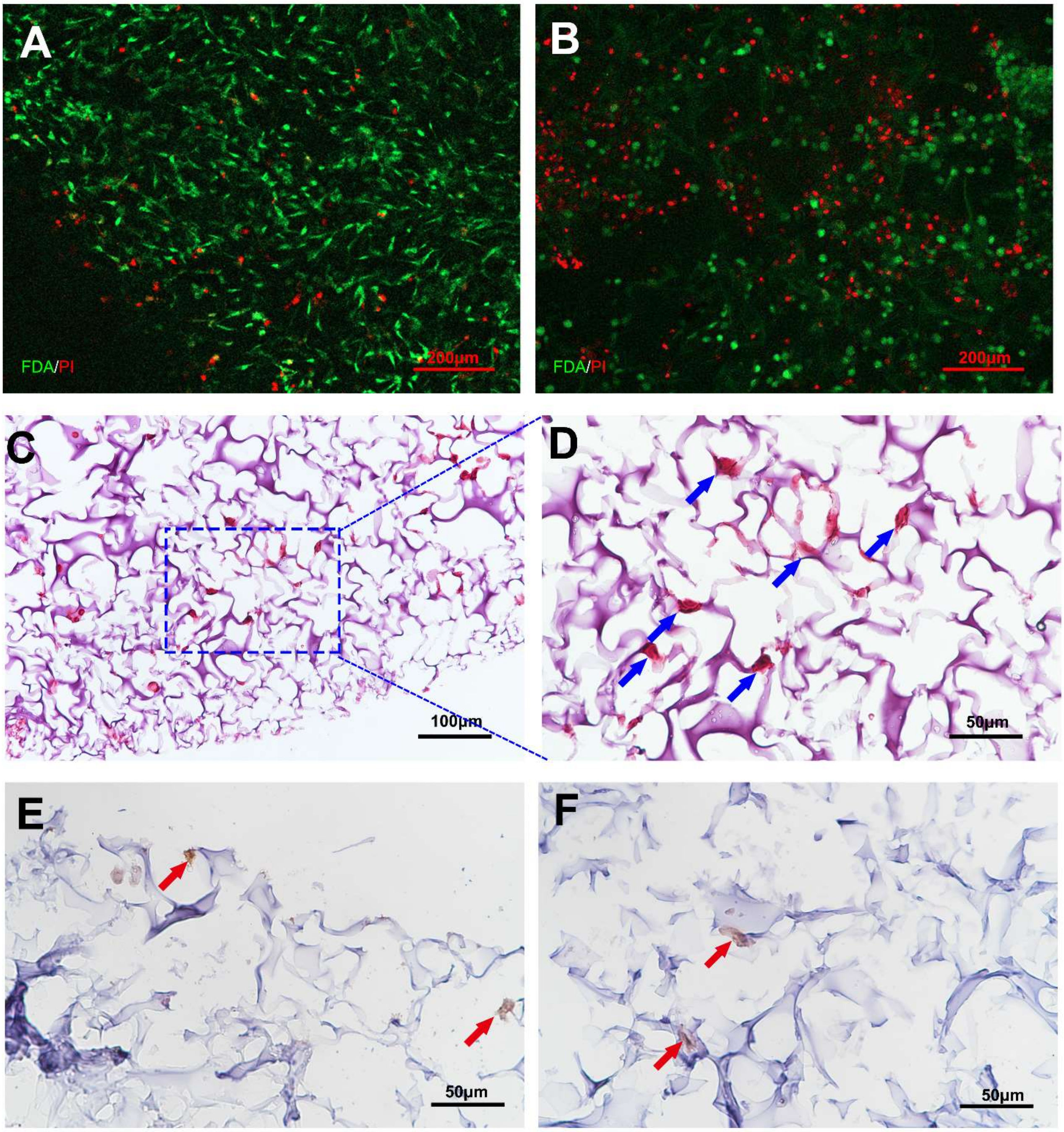

Figure S 3: The osteogenesis process of BMSCs.

BMSCs in gelatin scaffolds were stained with FDA/PI to confirm that the cells had successfully adhered to gelatin scaffolds (A, B). After a 21-days osteogenic induction, Alizarin red staining (C, D) and osteocalcin (OCN) immunohistochemistry staining (E, F) were conducted to confirm the osteogenic differentiation of BMSCs.
